# The impact of considering different numbers of contributors in identification problems involving real casework mixture samples

**DOI:** 10.1007/s00414-025-03500-7

**Published:** 2025-05-09

**Authors:** Camila Costa, Carolina Figueiredo, Sandra Costa, Paulo Miguel Ferreira, António Amorim, Lourdes Prieto, Nádia Pinto

**Affiliations:** 1 Faculdade de Ciências, Universidade Do Porto, Porto, Portugal; 2 i3S - Instituto de Investigação E Inovação Em Saúde, Universidade Do Porto, R. Alfredo Allen 208, 4200 - 135 Porto, Portugal; 3 Biologia, Laboratório de Polícia Científica da Polícia Judiciaria (LPC-PJ), Lisbon, Portugal; 4 IPATIMUP - Instituto de Patologia E Imunologia Molecular da Universidade Do Porto, Porto, Portugal; 5 Grupo de Medicina Xenómica, Instituto de Ciencias Forenses, Universidad de Santiago de Compostela, Santiago de Compostela, Spain; 6 Laboratorio ADN, Comisaría General de Policía Científica, Madrid, Spain; 7 CMUP, Centro de Matemática da Universidade Do Porto, Porto, Portugal

**Keywords:** NoC, Estimation, Forensic DNA, Likelihood Ratio, Weight-of-Evidence

## Abstract

Increasingly complex genetic samples are analyzed in forensic genetics routine, including mixtures to which more than one individual contributed. The standard problem relies on identification, aiming to quantify the likelihood of the donor of a reference sample being a contributor to the mixture. This is computed through a likelihood ratio (LR) and requires using devoted probabilistic genotyping software that may consider the quantity of the mixture’s DNA (quantitative tools), beyond only the presence/absence of specific alleles (qualitative tools). In any case, the mixture’s number of contributors (NoC) is a parameter that the user must introduce. Due to its nature, NoC is unknown for most real casework samples and needs to be estimated, which may be challenging due to poor DNA quality and quantity. This study aims to evaluate the impact of considering different NoC of real mixture samples (both over- and underestimating it after a first assessment of the expert) in identification problems through the pairwise comparison of LRs, using for the statistical assessment of both qualitative (LRmix Studio) and quantitative tools (EuroForMix and STRmix™). Different computational models showed different variations of the results, but for all, the impact was greater when considering a smaller NoC than the one initially estimated by the expert. Quantitative tools showed more sensitivity to NoC variation. Taking advantage of using real data, whose possible complexities surpass those of mock ones, this work highlights the impact that the NoC may have on the quantification of the proof, reinforcing the importance of its proper estimation.

**Supplementary Information:**

The online version contains supplementary material available at 10.1007/s00414-025-03500-7.

## Introduction

Forensic DNA samples recovered from the environment and for which more than one individual contributed have several characteristics that make them highly complex and difficult to interpret and analyze. Typically these samples are analyzed in the framework of identification problems, for which probabilistic genotyping methods were developed and implemented as informatic tools allowing the quantification of the weight of the evidence considering both the evidence and corresponding reference profiles [1, 2]. These tools can be based on two different models, either considering only the observed electropherogram’s peaks – qualitative model – or also considering their heights – quantitative model [3, 4]. On the other hand, the latter may be based on either maximum likelihood (numeral optimization), as [5], or random sampling (as Markov Chain Monte Carlo – MCMC), as [6], statistical methods. In any case, the weight of the evidence is measured through a Likelihood Ratio (LR) computation [7, 8] comparing the probability of observing the genetic evidence assuming two alternative and mutually exclusive hypotheses. This calculation also depends on several parameters regarding population and analytical factors that may need to be introduced by the user as a parameter in the software. This work focuses on the number of contributors (NoC), highlighting and reinforcing the crucial role that the expert continues to have in quantifying the proof as the results provided by the previously mentioned software highly depend on that parameter [9].

By nature, the NoC of most of the evidence samples analyzed in a forensic genetics routine is unknown and needs to be estimated, which can be a challenging task due to a. allele sharing between contributors, b. contributions in different proportions, c. stutter peaks which may be confused with alleles of a minor contributor (or vice-versa), or even d. amplification stochastic effects (heterozygotic imbalance, drop-in, and/or drop-out) due to low quantity and quality of DNA. Indeed, the decision concerning the presence (or not) of non-allelic peaks in the evidence sample [10–12] and the estimation of its NoC relies on the expert’s evaluation, which can be subjective and prone to errors [13, 14]. Experts may have different interpretations, especially in the most challenging samples [15]. The standard method for estimating NoC in forensic laboratories is based on the maximum allele count (MAC) observed at the locus with more alleles. A lower bound for NoC is calculated as half of the MAC from the autosomal locus (or loci) with more alleles [7, 13]. This initial estimate should then be re-evaluated by considering the peak imbalance in the electropherogram. In all instances, the subjective nature of MAC as an expert estimation must be acknowledged, as true alleles can be difficult to distinguish from stutters and other artifacts, especially in mixtures. Nevertheless, some informatics tools provide insights into which NoC better fits the information in the evidence electropherogram [16–20], by specifically targeting that variable. In other tools, the NoC is summed out [21].

Some studies have analyzed the impact of an incorrect NoC estimation on the LR value assigned by both qualitative and quantitative tools considering mock profiles [22–25]. The magnitude of the impact showed to be highly dependent on the analyzed samples, as an incorrect estimation can either have no significant effect or significantly decrease the LR value, favoring in some cases the alternative hypothesis of no contribution of the Person of Interest (PoI).

The main goal of this work is to analyze this impact by considering real casework samples with an unknown NoC, instead of measuring it in mock samples where the NoC is known. This methodology allowed to reliably replicate as much as possible the level of complexity of the conditions frequently encountered in the forensic genetics routine, which cannot be completely achieved with mocked samples. For each case, a NoC was estimated by the expert (eNoC) and then the over (NoC = eNoC + 1), and underestimation (NoC = eNoC- 1) of the value were also considered in the computations for comparison purposes. These samples were analyzed in both qualitative (LRmix Studio v.2.1.3 [26]) and quantitative informatics tools (EuroForMix v.3.4.0 [5] and STRmix™ v.2.7 [6]), and the results were compared in an intra-software analysis guaranteeing that the found variations are not due to the different statistical and computational architectures of the tools.

## Material and methods

Resorting to evidence material from former cases of the Portuguese Scientific Police Laboratory, Judiciary Police, 152 irreversibly anonymized pairs of samples were selected and analyzed, each one composed of a mixture with either two (eNoC = 2, N = 75) or three (eNoC = 3, N = 77) estimated contributors and a single-source profile associated – hereafter referred to as “reference sample”. The genetic information obtained for each pair of samples analyzed relies on a set of 21 autosomal short tandem repeat (STR) markers.

For each pair of samples, a statistical evaluation of the evidence was computed through LRs, under the framework of an identification problem, assuming the alternative hypotheses: “The PoI is a contributor of the mixture” and “The PoI is unrelated to any contributor of the mixture”. The reference sample was assumed to belong to the PoI. The LR values were assigned through both qualitative (LRmix Studio v.2.1.3 [26]) and quantitative (EuroForMix v.3.4.0 [5] and STRmix™ v.2.7 [6]) informatic tools. As the qualitative tool does not allow the inclusion of stutter peaks in the evidence analysis, this artifact was removed from the input file before any LRmix Studio computation, this being the only difference between the input genetic data considered in the tools. For all the calculations, the allele frequencies of the National Institute of Standards and Technology database concerning the Caucasian population were used [27] – see Online Resource 1. The software parameter values considered in the three tools were maintained as similar as possible and are described in Online Resource 2. Nevertheless, it should be remarked that these tools incorporate and apply different models to the input data, and as such, it will impact corresponding results. Regarding the inference approach of each tool, LRmix Studio uses the Maximum Likelihood Estimation (MLE) method, EuroForMix incorporates both MLE and integration (Bayesian) approach, and STRmix™ is based on a Bayesian approach using MCMC. In this work, the MLE approach was employed in EuroForMix as suggested by software developers for model comparison purposes [28]. Also, the parameter optimization is done differently, in both LRmix Studio and EuroForMix, a separate maximum likelihood is established for each of the abovementioned hypotheses, while in STRmix™, this optimization is done through the MCMC method. Focusing on the quantitative tools, even though both consider the quantitative information of the evidence’s EPG (i.e., the height of the peaks), the models implemented on them to analyze this information are quite distinct. EuroForMix applies a gamma function to model peak height distribution, while STRmix™ uses a log-normal one. Also, the artifacts present in the evidence profile as drop-in alleles and stutter peaks are modeled differently. The first is modeled in EuroForMix through an exponential function, while in STRmix™ is capped and can be modeled by either a Gamma (ɣ) or Uniform distribution, the latter having been considered in this work. Lastly, in EuroForMix the stutter peaks are modeled using a blanket ratio of all alleles, while in STRmix™, allele-specific ratios are applied.

To evaluate the impact in the weight of the evidence of considering different NoCs, cases with mixtures with two initially estimated contributors (eNoC = 2) were also analyzed as having three (NoC = eNoC + 1), while those with three initially estimated contributors (eNoC = 3) were analyzed as having four (NoC = eNoC + 1) and two (NoC = eNoC- 1) contributors.

For each tool's set of results, an intra-software analysis was performed by comparing the (non-null) LR value calculated under the default (LR_NoC=eNoC_; initial NoC estimation performed by the expert, eNoC) and the varied conditions ((LR_NoC=eNoC±1_: either NoC = eNoC + 1, or NoC = eNoC- 1), through the ratio between the two LR values: R = LR_NoC=eNoC_/(LR_NoC=eNoC±1_ if LR_NoC=eNoC_ > (LR_NoC=eNoC±1_, or R = (LR_NoC=eNoC±1_/LR_NoC=eNoC_, otherwise. A single run was performed for each software, pair of samples, and parameter conditions, except when the informatics tool was not able to make compatible the eNoC introduced by the user with marker-specific genotypic data, reporting either a null LR or an error message. For those cases, the marker-specific data that led to that output were withdrawn for comparison purposes, and LRs were recomputed, similar to that carried out in a previous study [9]. Alternatively to removing markers for which a null LR value was retrieved, software developers also suggest the variation of MCMC parameter values to test a possible convergence [29]. Indeed, due to the underlying stochasticity, null LRs could be a result of the lack of convergence of the method on which the software is based, the MCMC algorithms failing to identify a genotype combination compatible with the reference profile, as similarly observed in other studies [29–31]. Nevertheless, to maintain the methodology of a single run for each pair of samples, for all the programs, the first option was selected and a smaller set of markers (varying between 18 and 20) for which a non-null LR was obtained for 17% and 9% of the cases analyzed in STRmix™ with mixtures with two and three estimated contributors, respectively. These pairs of samples are clearly identified in Online Resource 3 by LR < 21 and will also be identified in all the visual representations of the data. Statistical significance of the results was reached considering a significance level α = 0.05.

## Results and discussion

**Fig. 1:**
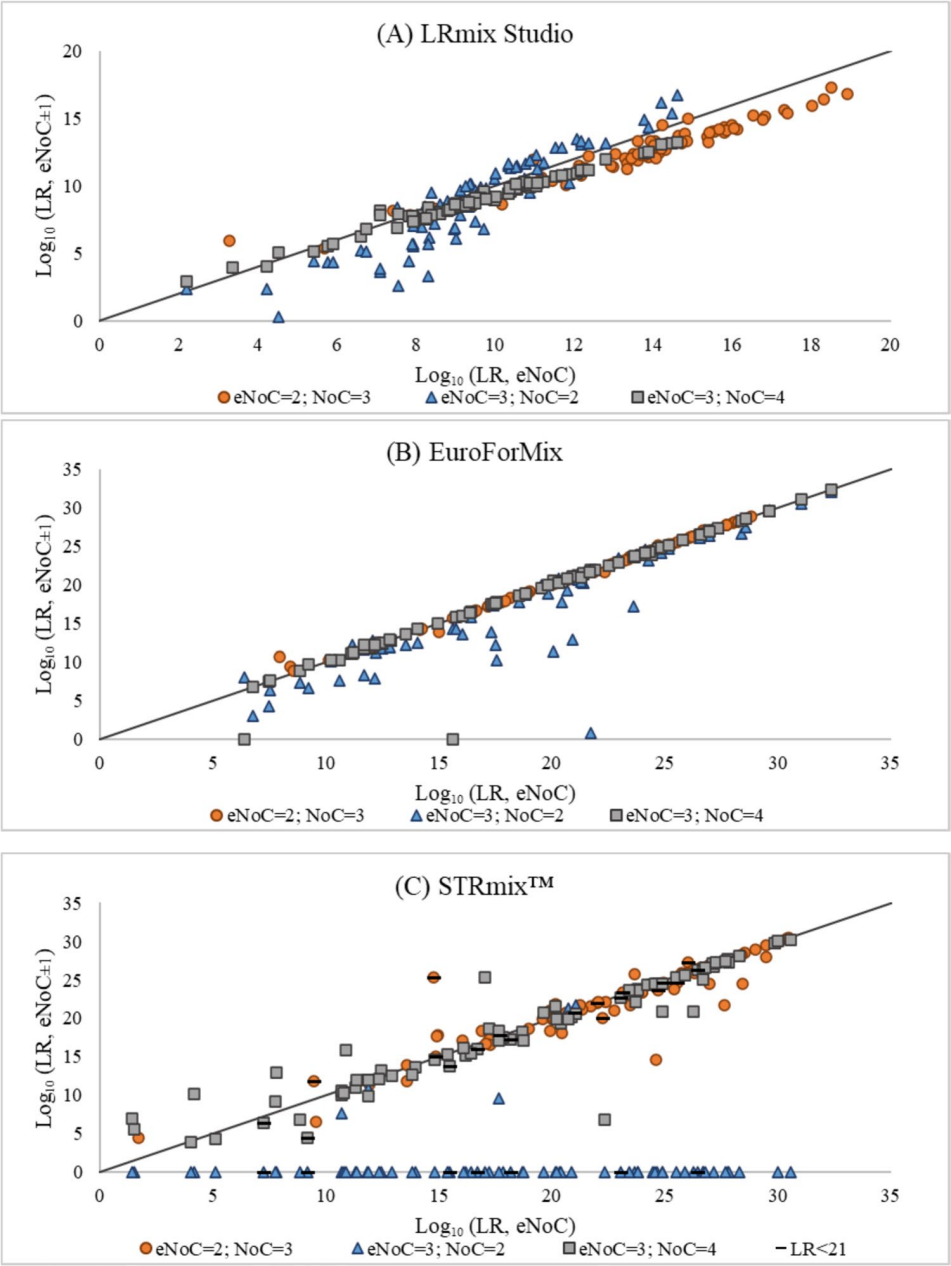
Distribution of LR values (log_10_ scale) assigned through LRmix Studio v.2.1.3 (A), EuroForMix v.3.4.0 (B), and STRmix™ v.2.7 (C), assuming the initial estimation of the expert for the number of contributors of the mixture (x-axis) for both cases with two and three estimated contributors and its variation (y-axis) – over (circles for eNoC = 2 and squares for eNoC = 3) and underestimation (triangles for eNoC = 3). The cases for each less than 21 markers were considered for STRmix™ are highlighted with a ‘- ‘ over the corresponding point

The LR values assigned by LRmix Studio v.2.1.3 [26], EuroForMix v.3.4.0 [5], and STRmix™ v.2.7 [6] assuming the initial estimation of the expert for the number of contributors of the mixture sample (eNoC, equal to either two or three) and the varied parameter (NoC = eNoC + 1: from two to three and from three to four, and NoC = eNoC- 1: from three to two) are displayed in Fig. 1.

Table 1 summarizes the log_10_ scale of the ratios (R) calculated between LR values computed assuming the initial estimation of the NoC (eNoC, equal to either two or three) and the varied parameter (NoC = eNoC + 1: from two to three and from three to four, and NoC = eNoC- 1: from three to two) for the three informatics tools considered. Broadly speaking these data show how many orders of magnitude the paired values: LR_NoC=eNoC_ and LR_NoC=eNoC±1_, differ between them. Consider as an example the first pair of samples (1_2) for which eNOC = 2 and the EuroForMix tool. Assuming both the eNoC value and its overestimation (NoC = 3) two LR values were computed: LR_NoC=2_ = 8.47E + 07, and LR_NoC=3_ = 5.13E + 10. Afterward, a ratio R > 1 between the two values was performed and considered for further analyses in a log_10_ scale: log_10_ (R = LR_NoC=3_/LR_NoC=2_) = 2.782. In Table 1, this case is then accounted for the 2 < R < 3 category, which means that baseline and varied LR values differ in more than two but less than three units on a log_10_ scale, i.e., the greatest LR is at least 100 times higher than the other but not overpassing 1000 times.

**Table 1:** Summary of the ratios (R, log_10_ scale) found between LR values calculated using the computer tools: LRmix Studio v.2.1.3, EuroForMix v.3.4.0 (EFM), and STRmix™ v.2.7, considering as NoC the number of contributors estimated by the expert (eNoC) and + 1 (NoC = eNoC + 1, from two to three and from three to four) and − 1 (NoC = eNoC- 1, from three to two). N/A: “Not Applicable” corresponds to situations where no comparisons were performed, either because an LR equal to zero was retrieved or an error message was displayed. ^a^ LR values assigned for less than 21 markers included (17% and 9% of the analyzed cases for eNoC = 2, and eNoC = 3, resp.). *See Online Resource 3 for more data. The statistical significance for the LR to increase or decrease when the NoC was considered as eNoC + 1 and eNoC- 1 is also presented (α = 0.05); significant values are underlined

Ratios between LR values (R, log_10_ scale)	Estimated NoC (eNoC) = 2			Estimated NoC (eNoC) = 3					
	** + **1			** + **1			**− **1		
	LR|NoC = 2 vs. LR|NoC = 3			LR|NoC = 3 vs. LR|NoC = 4			LR|NoC = 3 vs. LR|NoC = 2		
	LRmix Studio	EFM	STRmix™	LRmix Studio	EFM	STRmix™	LRmix Studio	EFM	STRmix™
**0 < R < 1**	31%	97%	67% ^a^	86%	97%	70% ^a^	49%	60%	9%
**1 < R < 2**	61%	1%	16% ^a^	14%	0%	14% ^a^	27%	21%	1%
**2 < R < 3**	8%	1%	11%	0%	0%	3%	14%	4%	0%
**3 < R < 4**	0%	0%	3%	0%	0%	0%	4%	6%	1%
**R > 4**	0%	0%	4% ^a^	0%	0%	13% ^a^	5%	9%	1%
**N/A**	0%	0%	0%	0%	3%	0%	0%	0%	87% ^a^
**The maximum ratios between LR values** (R, log_10_ scale)									
** R**	2.713	2.782	10.520	1.333	0.508	15.510	6.356	20.929	8.059
** LR|NoC = eNoC**	1.83E + 03	8.47E + 07	5.58E + 14	2.43E + 14	2.69E + 24	2.10E + 22	2.19E + 03	5.40E + 21	4.45E + 17
** LR|NoC = eNoC ± 1**	9.44E + 05	5.13E + 10	1.85E + 25	7.51E + 13	8.35E + 23	6.52E + 06	9.65E- 04	6.36E + 00	3.88E + 09
Pair*	1_2	1_2	8_2	77_3	49_3	51_3	17_3	5_3	44_3
**LR tends to (α = 0.05)**									
	Decrease	Increase	Decrease	Decrease	Increase	Decrease	-	Decrease	-
**p-value**	3.47E- 13	6.69E- 06	7.91E- 03	3.61E- 12	4.96E- 02	2.48E- 05	9.09E- 01	2.48E- 05	5.27E- 01

The corresponding trend for the LR to increase or decrease its value when the NoC was varied is also shown in Table 1. See Online Resource 3 for the complete set of LR results assigned by the three software under the tested conditions.

**Fig. 2:**
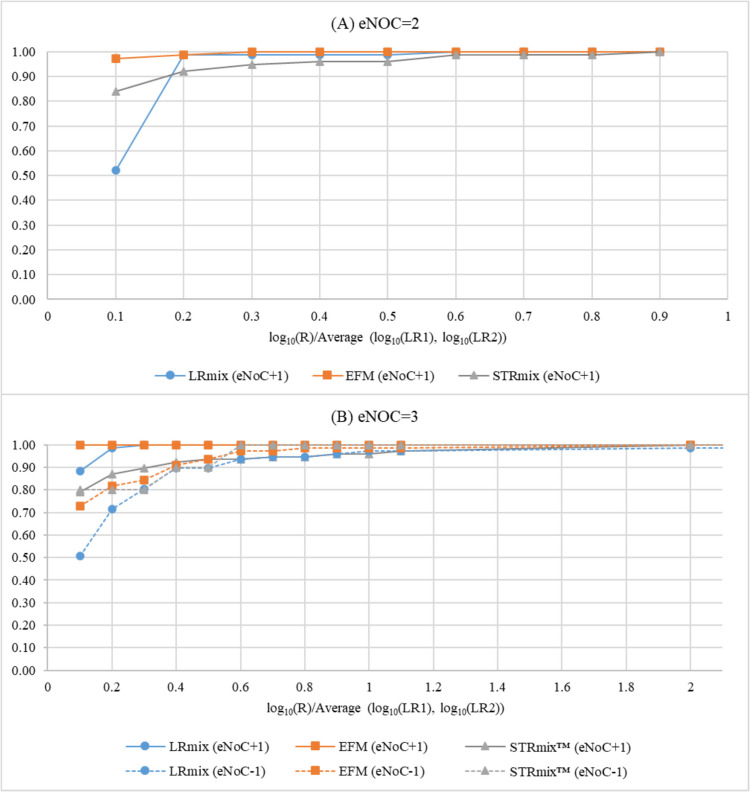
Cumulative distribution of the differences between LR values assigned through LRmix Studio v.2.1.3 (circles), EuroForMix v.3.4.0 (EFM; squares), and STRmix™ v.2.7 (triangles), normalized by their average, for both cases with mixtures with two (eNoC = 2; N = 75; (A)) and three (eNoC = 3; N = 77; (B)) estimated number of contributors, when NoC was either over- (continuous line) or underestimated (dashed line). Data regarding the outlier sample pair 17_3 and the software LRmix Studio was excluded due to scale limitations (x = 39.10) – see main text and Table 2 for more details

To illustrate how much the values have changed relatively to their size, Fig. 2 presents the cumulative distribution of the differences existing between the baseline (LR_NoC=eNoC_, log _10_ scale) and the varied values (LR_NoC=eNoC±1_, log_10_ scale), normalized by their average. For example, for the previously mentioned case 1_2 the computed value equated: log_10_(R = LR_NoC=3_/LR_NoC=2_)/[(log_10_(LR_NoC=3_) + log_10_(LR_NoC=2_))/2] = 2.782/9.319 = 0.299. Assuming this approach, the most striking results were observed for cases with mixtures for which eNoC = 3 – see Table 2. From the cases presented in Table 2, special attention should be given to pair 17_3 which was not represented in Fig. 2 due to scale constraints, and that had already been identified in Table 1 as one of those with the greatest R values. This was the only case for which the variation of NoC led to an LR value smaller than 1, supporting the alternative hypothesis of unrelated individuals. Recognizing the qualitative nature of the LRmix Studio software, inspecting the genotypic data of the pair 17_3 supports its inability to justify via drop-in the high number of peaks present in the evidence sample when underestimating the NoC. Under the same assumptions, quantitative software may have considered other possibilities, such as stutters, to interpret and quantify the genetic evidence. Still, regarding the sample pairs mentioned in Table 2, all the other differences observed for the quantitative tools were due to large partial LR variations in a small (two or three) set of markers, where the height of the peaks severely impacted the likelihood of the observations assuming the hypotheses, allele frequencies and parameter values.

**Table 2:** Detailed information regarding the most striking results observed (> 1) when the differences (log_10_(R)) between LR values (assigned assuming both default – eNoC – and varied condition – eNoC ± 1) were normalized by their average, only observed for cases with mixtures with three initially estimated number of contributors (eNoC = 3; N = 77)

Varied condition	Informatics tool	Pair	LR (default; eNoC = 3)	LR (varied; eNoC ± 1)	Log_10_(R)	Average	Normalization
**eNoC + 1**	**STRmix™**	12_3 15_3 51_3	3.32E + 01 2.76E + 01 2.10E + 22	4.45E + 05 9.75E + 06 6.52E + 06	4.127 5.549 15.509	3.585 4.215 14.568	1.151 1.317 1.064
**eNoC- 1**	**LRmix Studio**	17_3 18_3	9.65E- 04 2.12E + 00	2.19E + 03 3.31E + 04	6.356 4.193	0.163 2.424	39.103 1.730
	**EuroForMix**	5_3	5.40E + 21	6.36E + 00	20.929	11.268	1.857

Differences higher than four log_10_ units ($\text{R}>\text{10,000}$) were obtained for all the software for eNoC = 2 and/or eNoC = 3. Special attention was devoted to some of these cases trying to disclose the motifs that may have led to such difference, and possible reasons are many fold. Overall, STRmix™ showed the greatest number of cases with $\text{R}>\text{10,000}$; however, the stochasticity of the methods on which this tool is based should not be disregarded as these differences may be due to the lack of convergence of MCMC algorithms rather than due to the NoC variation itself. Other reasons for this and the remaining tools may include the possibility of CE failure to correctly attribute the alleles, errors in experts’ analysis of evidence samples (selection of true alleles), highly degraded samples, or even, incorrectly assigning eNoC. In any case, as referred to in Material and Methods section, the same input files were used for the three tools, showing that different software are more sensitive to some variations than the others, depending on samples’ characteristics.

### *a) When considering* + *1 contributor than those estimated by the expert*

For the three tools, a generally greater impact was observed for cases with mixtures with eNoC = 2 compared to those for which eNoC = 3, which may be due to the smaller complexity of the first (i.e. fewer possibilities of contributors), as observed in previous studies [9, 32]. In any case, it should be noted that the most extreme results, when normalized by the mean values, were obtained for cases with mixtures with eNoC = 3, as shown in Fig. 2 and Table 2.

For the qualitative tool LRmix Studio, most differences were between one and two units (log_10_ scale): $10<\text{R}<100$, for cases with mixtures with eNoC = 2, but did not overstep one unit for most cases with eNoC = 3: $1<\text{R}<10$.

The smallest differences observed were in fact for the quantitative tool EuroForMix, as in almost all cases, differences lower than one unit were obtained: $1<\text{R}<10$. Nevertheless, for 3% of cases with mixtures with eNoC = 3, an error message popped up when considering NoC = 4. These cases are represented in Table 1 as N/A and were not considered in the analysis.

On the other hand, only for the other quantitative software STRmix™ differences higher than four units (i.e., $\text{R}>\text{10,000})$ were obtained, for both cases eNoC = 2 and eNoC = 3. Those differences resulted indiscriminately from either the decrease or increase of the LR value. The maximum differences achieved in STRmix™ were much higher ($\text{R}={10}^{10.520}$ and $\text{R}={10}^{15.510}$) than those obtained for LRmix Studio ($\text{R}={10}^{2.713}$ and$\text{R}={10}^{1.333}$) and EuroForMix ($\text{R}={10}^{2.782}$ and$\text{R}={10}^{1.262}$), for both cases with eNoC = 2 and eNoC = 3, respectively.

Both LRmix Studio and STRmix™ showed a statistically significant trend for lower LR values when NoC = eNoC + 1, for both types of mixtures with eNoC = 2 and eNoC = 3 (Table 1), similar to previous studies performed with mock samples and known NoC [22, 24]. The decrease in the LR value can be associated with the increase in the number of possible combinations of genotypes when assuming one more contributor than initially considered. EuroForMix software showed the opposite trend – i.e., LR values tend to be higher when the NoC = eNoC + 1, being also noteworthy that it was the tool with the greatest proportion of cases differing in less than one order of magnitude (97% of the cases for both eNoC = 2 and eNoC = 3) that supports a modest practical impact of the NoC variation. In any case, several considerations should be made regarding these results on the increase/decrease of the LR values depending on the NoC. First, it should be borne in mind the unpredictable nature and unknown composition of the analyzed data as, contrarily to what occurs for mocked samples, it is impossible to be sure regarding the true NoC, and even if the profile is considered as a reference truly contributed to the evidence. Moreover, and in any case, caution should be taken when comparing the LR values obtained by the different tools to evaluate the impact of the different statistical and computational models, not only because STRmix™ is based on a stochastic architecture and a single run was performed for each case, but also because the number of markers analyzed were not always the same, as previously explained and clearly identified in Online Resource 3. For example, for the previously referred pair 1_2, STRmix™ LR values were computed considering 18 out of the 21 markers, as three of them retrieved marker-specific LR values equal to zero under the default condition (eNoC = 2), having been removed for further analyses. For the other tools, the complete set of 21 markers was considered as none retrieved an LR equal to zero.

### *b) When considering − 1 contributor than those estimated by the expert*

As abridged in Table 1, the majority of the differences observed were lower than one unit (log_10_ scale) for all the informatics tools: $1<\text{R}<10$. Yet, differences higher than four units (log_10_ scale): $\text{R}>\text{10,000}$ were also found. The maximum difference obtained for both LRmix Studio and STRmix™ ($\text{R}={10}^{6.356}$ and $\text{R}={10}^{8.059}$, respectively) was much lower than the one assigned by EuroForMix ($\text{R}={10}^{20.929}$).

Nevertheless, these data must be taken with care, and it should be borne in mind that for most STRmix™ calculations (87%) no comparisons were performed since the software was not able to conciliate the number of NoC = eNoC- 1 with the genotypic data – in 74% of cases, the software was unable to assign an LR value presenting an error message, and in 13% it retrieved an LR equal to zero. These cases are represented in Table 1 as N/A and were not considered in the following analysis.

For the remaining STRmix™ results, there was no evidence of a trend for LR value to increase or decrease when the NoC = eNoC- 1. An analogous situation was verified for LRmix Studio. Only for EuroForMix results, a statistically significant trend was observed, as the LR decreased when considering one less contributor than initially estimated. This decrease may be a consequence of low-height alleles in the mixture (corresponding to the reference sample) being considered a stutter or even a non-specific peak to enable the tool to deal with the existence of more peaks per marker than would be expected for the estimated NoC. Since EuroForMix has a more tolerant peak variance parameter it allows a higher rate of false support of non-contributors [30]. Therefore, in most cases, the software assigned an LR value but a smaller one. Yet, for some others, it could not justify the presence of so many peaks per marker given the NoC proposed resulting in LR values lower than one. In any case, and as previously explained, caution should be taken in the generalization and interpretation of these results.

## Conclusions

The ability to analyze highly complex DNA forensic data leads to the corresponding statistical evaluation being dependent on the use of informatic tools that consider several parameters of interest, such as the number of contributors (NoC) of the evidence sample. This parameter is usually unknown and needs to be estimated by the forensic expert on a case-by-case basis. This analysis may be subjective particularly when dealing with complex samples with poor quality and quantity DNA, as is the case of some real casework samples as those considered in this work. It is thus fundamental to understand to what extent different values of the NoC may affect the statistical evaluation of the genetic evidence. In this study, 152 pairs of real casework samples composed of a mixture (with the estimated number of contributors– eNoC – equal to either 2 or 3) and a reference sample were analyzed through different tools, two taking into consideration the quantity of DNA (EuroForMix, and STRmix™, quantitative tools) and one disregarding such information (LRmix Studio, qualitative tool). For each pair of samples, the LR value assigned assuming the initial estimation eNoC was compared with the one assigned assuming + 1 (for both cases with mixtures with eNoC = 2 and eNoC = 3) and − 1 (only for eNoC = 3). In the first case: NoC = eNoC + 1, a trend for LR decreasing was observed for two tools (LRmix Studio and STRmix™ comparisons), while for EuroForMix the opposite trend was verified. LR discrepancies greater than 10,000 × were only found for one of the quantitative tools: STRmix™, for both eNoC = 2 and eNoC = 3. On the other hand, when considering NoC = eNoC- 1, LR differences greater than 10,000 × were verified for all the analyzed tools, but a statistically significant trend was only found for one of the quantitative tools: EuroForMix, specifically for LR values decreasing. Comparing the impact of considering eNoC + 1 and − 1, the latter seems to have a greater impact on all the informatics tools, being more emphasized in quantitative ones (STRmix™ and EuroForMix). Even so, STRmix™ stands out, since for 87% of the complete set of cases no comparisons were performed, showing to be more strict in the analysis of considering the eNoC incompatible with the genotypic data than the other two. When normalizing these values by the corresponding average, the most striking LR differences were obtained for cases involving mixtures with eNoC = 3. A limitation of the study is that LRs for non-contributors were not included.

Developing research using real casework data is a straightforward and needed way to better understand and approximate what practitioners encounter in the casuistic routine as they enclose complexities unable to be predicted or replicated in mocked samples. Recognizing its added value, the nature of the samples analyzed should not be however overlooked and caution should be taken when generalizing and interpreting the obtained results as the challenges are many folds. For example, given the unknown composition of the data, it is impossible to be sure of both the true number of contributors of the evidence samples and if the associated single-source ones contributed to them, which may compromise general conclusions regarding the increase or decrease of LR values depending on the over- or underestimation of the NoC. In fact, this work supports that the impact of varying the NoC parameter in the computed LR is highly variable, depending on both the genotypic profiles of the samples and the computational approach used to accommodate all the parameters.

Even though probabilistic genotyping tools assist the expert in the statistical analyses of complex forensic DNA samples, several parameter values must be introduced in the software before performing any computation, such as the NoC of the evidence samples under analysis. This work emphasizes and demonstrates that different NoC values may have crucial implications in the computation of the weight of the proof, reinforcing the importance of critically analyzing the outcome provided by statistical tools..

## Supplementary Information

Below is the link to the electronic supplementary material.

Supplementary file1 (XLSX 13 KB)

Supplementary file2 (DOCX 16 KB)

Supplementary file3 (XLSX 50 KB)

## Data Availability

All the statistics that supported the results presented in this study are provided in the Supplementary Material for each one of the analyzed pairs of samples and parameters. The raw genotypic and haplotypic analyzed data concern real forensic casework from the Portuguese Scientific Police Laboratory, Judiciary Police (LPC-PJ), and for that reason, its release is not possible. In any case, specific data from specific analyzed markers (non-haplotypic data) may be available from the authors upon reasonable request and with the permission of LPC-PJ. The summary statistics of specific runs can also be provided to the readers when solicited. For data sharing issues, contact the corresponding author of the manuscript.
