# Supplementary material for: The impact of considering different numbers of contributors in identification problems involving real casework mixture samples
Source: Int J Legal Med. 2025 May 9;139(5):2099–107. doi: 10.1007/s00414-025-03500-7 (PMC12354499; doi:10.1007/s00414-025-03500-7)
Supplement: Supplementary file 2 — Supplementary file2 (DOCX 16 KB) [file 414_2025_3500_MOESM2_ESM.docx]

**Table ESM_2**: Software parameter values introduced in the three informatics tools: LRmix Studio v.2.1.3, EuroForMix v.3.4.0, and STRmix^TM^ v.2.7. ^a^ Dropout is directly estimated through the peak height distribution. ^b^ Per locus, specified by the software considering the default conditions (i.e. disregarding the population size).

| **Values**  **Parameters** | **LRmix Studio** | **EuroForMix** | **STRmix^TM^** |
| --- | --- | --- | --- |
| **Coancestry Coefficient** | 0.01 | | |
| **Drop-in frequency** | 0.05 | | |
| **Drop-in parameters’ distribution** | N/A | Lambda (λ):  0.01 | Gamma (ɣ): (0.1,0.1) |
| **Drop-in cap** | N/A | N/A | 100 |
| **Dropout** | 0.1 | ^a^ | ^a^ |
| **Minimum allele frequency** | 0.001 | 0.001 | ^b^ |
| **Threshold detection** | N/A | 100 | 100 |
| **Stutters** | Without | With | With |
